# Bradycardic and Antiarrhythmic Effects of the D-Limonene in Rats

**DOI:** 10.5935/abc.20190173

**Published:** 2019-11

**Authors:** Gildenisson Araújo do Nascimento, Diego Santos de Souza, Bruno Santos Lima, Carla Maria Lins de Vasconcelos, Adriano Antunes de Souza Araújo, Aimée Obolari Durço, Lucindo José Quintans-Junior, Jackson Roberto Guedes da Silva Almeida, Aldeída Pereira Oliveira, Valter Joviniano de Santana-Filho, André Sales Barreto, Márcio Roberto Viana dos Santos

**Affiliations:** 1 Universidade Federal de Sergipe, São Cristóvão, SE - Brazil; 2 Universidade Federal do Vale do São Franscisco, Petrolina, PE - Brazil; 3 Universidade Federal do Piauí, Teresina, PI - Brazil

**Keywords:** Rats, Limonene, Monoterpenes, Arrhythmias, Cardiac, Anti-Arrhythmia Agents, Bradycardia, Antioxidants

## Abstract

**Background:**

D-limonene (DL) is a monoterpene and is the major component in the essential oil of citrus fruit. It presents antihyperglycemic and vasodilatation activities.

**Objectives:**

This study evaluated the cardiovascular effects and potential antiarrhythmic of DL in rats.

**Methods:**

Hemodynamic and electrocardiographic (ECG) parameters were measured in male Wistar rats, which under anesthesia had been cannulated in the abdominal aorta and lower vena cava and had electrodes subcutaneously implanted. In the in vitro approach, the heart was removed and perfused using the Langendorff technique. The significance level adopted was 5% (p < 0.05).

**Results:**

DL, in doses of 10, 20, and 40 mg/kg (i.v), produced intense and persistent bradycardia associated with hypotension. Bradycardia with prolonged QTc was observed in the ECG in vivo recording. In the in vivo model of arrhythmia induced by Bay K8644, DL (10 mg/kg) decreased the arrhythmia score from 15.33 ± 3.52 to 4.0 ± 2.64 u.a (p < 0.05, n = 4). In isolated perfused hearts, DL (10^-3^ M) promoted significant reductions in heart rate (from 228.6 ± 8.5 ms to 196.0 ± 9.3 bpm; p < 0.05) and left ventricular development pressure (from 25.2 ± 3.4 to 5.9 ± 1.8 mmHg; n = 5, p < 0.05).

**Conclusions:**

DL produces bradycardia and antiarrhythmic activity in rat heart.

## Introduction

Despite the extensive research carried out on cardiac arrhythmias over the past decades, only a small number of antiarrhythmic drugs have emerged to add to the current therapeutic arsenal for this condition. Furthermore, "classical" antiarrhythmic drugs have serious efficacy and safety limitations and present several side effects.^1,2^

In the search for therapeutic alternatives for arrhythmias, recent studies have screened naturally occurring chemical compounds aiming to evaluate their effects on cardiac parameters. For example, studies with the monoterpenes pulegone and geraniol showed a negative inotropism in the mammalian myocardium through blockage of the Ca^2+^ and K^+^ currents.^3-5^ Most recently, Vasconcelos et al.^6^ verified that citronellol and nerol present negative inotropism on the left atrium of guinea-pigs.

**Figure 1:**
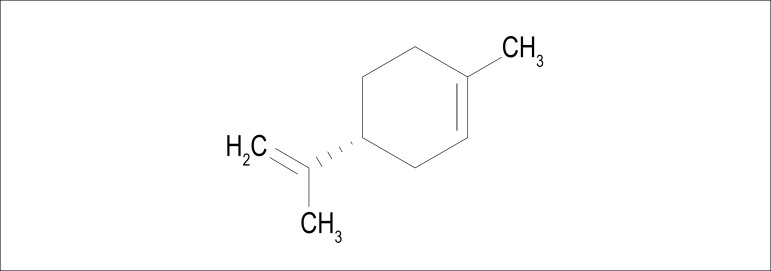
Chemical structures of the d-limonene (DL) (FW: 136.24).

D-limonene (DL) (Figure 1), is an alcoholic monoterpene and the major constituent of the essential oil extracted from citrus species such as lemon, lime and orange.^7^ Previous studies have demonstrated that DL has low toxicity and presents antioxidant,^8,9^ vasodilatation,^10,11^ and antihypertensive proprieties.^12^ The effect of DL on the heart has not yet been elucidated and investigations into its action mechanism can provide new insights about DL and the cardiovascular system. The current study, therefore, aimed to evaluate the potential antiarrhythmic effect de DL using both *in vivo* and *ex vivo* approaches.

## Methods

### Chemicals

The chemicals used were: d-limonene (DL: purity: 97%), and S-(−)-Bay K8644, both purchased from Sigma Aldrich (Chemical CO, USA), and ketamine and xylazine (purchased from Sespo, São Paulo, Brazil). The DL stock solution was prepared using a saline/cremophor mixture (0.15% v/v), the S-(−)-Bay K8644 was diluted in methanol and the DL solution in distilled water. Stock solutions were preserved at 0ºC and diluted with distilled water, if necessary. The vehicles (methanol or saline+cremophor) revealed no effect on the control preparations.

### Animals

Thirty-six healthy rats (*Rattus norvegicus*), weighing between 250 and 350 g were obtained from colonies from the Physiology Department of the Federal University of Sergipe, Brazil. They were maintained under standard temperature conditions (22 ± 2ºC) on a 12-hour light/dark schedule (lights on at 06 am) and were fed a standard diet (Labina®) with water *ad libitum*.

In each set of experiments, rats were divided into two groups: control with 4 or 6 animals, and DL treated, with 4 or 6 animals. All experimental protocols were previously approved by the Animal Care and Ethics Committee of the Universidade Federal de Sergipe.

### Effects of DL on Hemodynamic parameters in vivo

Rats were anesthetized with ketamine and xylazine (80 mg/Kg and 10 mg/Kg; i.p., respectively), and polyethylene catheters were implanted in the abdominal aorta and inferior vena cava via left femoral artery and vein, respectively. After insertion and fixation by cotton threads, the catheters were tunneled subcutaneously and exteriorized through an incision in the posterior cervical region of the animal. The incisions were closed and the animals were allowed a 24-hour post-operative recovery period. Mean arterial pressure (MAP) and heart rate (HR) were recorded through a pressure transducer (Edwards LifeSciences, Irvine, CA, EUA) coupled to an amplifier (FE221, Bridge Amp; ADInstruments, Bella Vista, NSW, Australia)

After post-operative recovery period, MAP and HR were recorded before (baseline values) and after IV bolus administration of DL (1, 5, 10, 20, and 40 mg/kg, i.v.) or vehicle to obtain dose-response curves. The DL curve (n = 6) was compared with the vehicle curve (n = 6).

### In vivo electrocardiographic measurements

Animals from both groups, control (n = 4) and DL treated (n = 4), were heparinized (200 I.U, i.p.), anesthetized with an i.p. injection of ketamine (80 mg/kg) and xylazine (10 mg/kg), and the vena cava was cannulated as previously described. The rats were kept in the supine position with spontaneous breathing and were administrated vehicle or DL (10 mg/kg). The volume injected through the cannula was the same for all concentrations (200 µL). To record the electrocardiographic signal, the electrodes were placed under the skin in the DII lead arrangement. The electrocardiographic parameters observed were the PR interval, QT interval, duration of QRS complex and HR. The QT interval was corrected by Bazett's formula modified for rats.^13^

### Isolated perfused heart (Langendorff technique) experiments

Animals, from both groups, control (n = 4) and DL treated (n = 4), were heparinized (200 I.U., i.p.) and after 15 min the heart was removed and mounted in an aortic perfusion system of the Langendorff type, on a constant flow (10 mL/min).^14^ The hearts were continuously perfused with Krebs-Henseleit solution (in mM: 120 NaCl, 5.4 KCl, 1.2 MgCl_2_, 1.25 CaCl_2_, 2 NaH_2_PO_4_, 27 NaHCO_3_, 11 glucose), previously filtered through a cellulose acetate membrane (0.45 µm), pH was adjusted to 7.4 and oxygenated (95% O_2_ + 5% CO_2_) and maintained at 37 ± 0.1ºC (Haake F3, Berlin, Germany). Electrocardiographic (ECG) heart signals were captured using three electrodes (Ag/AgCl/NaCl, 1 M) that were placed inside the chamber close to the heart. The signals were amplified, digitalized (PowerLab 4/35 ADInstruments, USA) and stored in a computer. Left ventricular development pressure (LVDP, mmHg) and HR (bpm) were measured using a water-filled balloon introduced into the cavity of the left ventricle. This device was coupled to a pressure transducer (FE221, Bridge Amp, ADInstruments, USA) and an amplifier (PowerLab 4/35, ADInstruments). The system was calibrated using a column of mercury. Time of peak (ms), which is defined as the time necessary to achieve the peak of maximal ventricular contraction, and relaxation time, were also analysed.

### Evaluation of the antiarrhythmic effect of DL

The antiarrhythmic effect of DL was determined the *in vivo* animal model using (S)-(−)-Bay K8644, an L-type calcium channel (LTCC) agonist. Arrhythmia was induced by administration of (S)-(−)-Bay K8644 (1 mg/kg, i.v.) in the femoral vein of both groups, control (n = 4) and DL treated (n = 4).

DL (10 mg/kg) was administered 10 min prior to (S)-(−)-Bay K8644.^15^ Cardiac arrhythmias were observed for 20 min by analysis of the ECG recording. For a better analysis, the total time was divided into 10, 3 min intervals for classification of arrhythmia. Arrhythmias were determined through a modified scoring system previously validated by Curtis and Walker.^16^ The arrhythmias observed were ventricular premature beats (VPB), ventricular tachycardia (VT) and ventricular fibrillation (VF). VPBs < 10/3 min period were recorded as 0; VPB’s > 10/3 min were recorded as 1; VT episodes of 1-5/3 min were recorded as 2; > 5/3 min episodes of VT and/or any number of episodes of VF and/or 1 episode of VF with a duration < 40 s period was recorded as 3; 2-5/3 min episodes of VF or a VT and VF with a total combined with duration < 80 s period was recorded as 4; 5/3 min episodes of VF or VT and VF combined with a duration < 160 s was recorded as 5; VT or VF or both/3 min with a total combined duration < 300 s were recorded as 6, and VT or VF or both/3 min with a total combined duration > 300 s were recorded as 7.

### Statistical analysis

The normality of the data was verified by using Shapiro-Wilk normality test. The data were represented as mean ± SD. When appropriate, it was used either unpaired Student T-test or analysis of variance (one or two way ANOVA) and Bonferroni’s post hoc test. The significance level adopted was 5% (p < 0.05). Statistical analyses were performed using GraphPad Prism™ 5.0.

## Results

**Figure 2:**
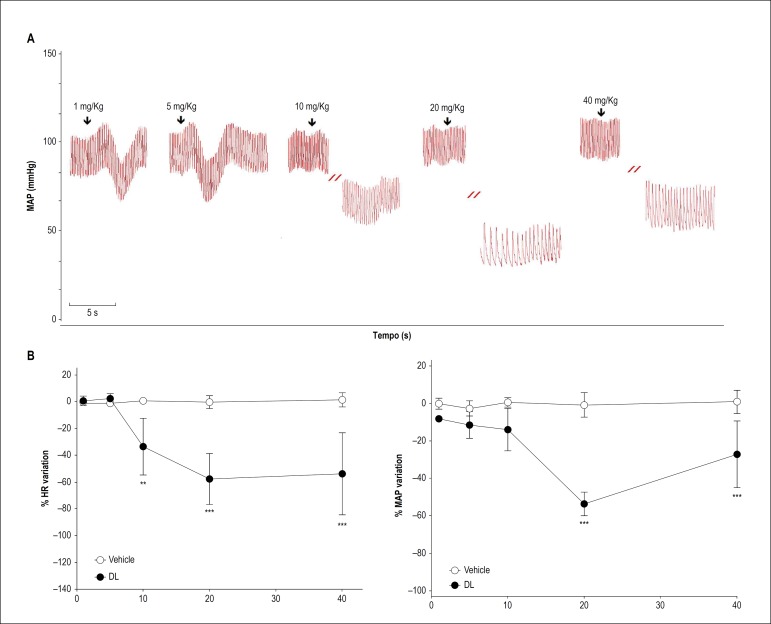
Effect of DL (1, 5, 10, 20, and 40 mg/kg, i.v.) on heart rate (HR) and mean arterial pressure (MAP) in conscious rats. A) Original traces showing the DL effect (1, 5, 10, 20 and 40 mg/Kg, i.v., randomly) on heart rate (HR) and mean arterial pressure (MAP) from one healthy and conscious rat. The arrows indicate the moment of administration. B) Line graph of DL effect on HR and MAP. Values are mean ± SD from six experiments. Data were analysed by repeated measures two-way ANOVA followed by Bonferroni post-test. ** p < 0.01 and *** p < 0.001 vs vehicle.

In all rats tested, HR and MAP baseline values were 371 ± 45 bpm and 114 ± 11 mmHg, respectively. Figure 2A shows original traces of the DL effect (1, 5, 10, 20, and 40 mg/kg, i.v.) on the pulsatile signal of arterial pressure of a conscious rat. In these animals, DL, at doses of 1 and 5 mg/kg, produced transitory and non-significant hypotension without altering HR (Figure 2B - left graph). On the other hand, DL, at doses of 10, 20, and 40 mg/kg, produced an intense and persistent bradycardia (-33.0 ± 21; -57.0 ± 19, and -53.0 ± 30 %; n = 6; p < 0.05, respectively) (Figure 2B - right graph) which persisted up to 19 ± 12 min after administration. This bradycardia was associated to hypotension only in the doses of 20 and 40 mg/kg (-53.0 ± 6, and -28.0 ± 18 %; n = 6, p < 0.05, respectively).

**Figure 3:**
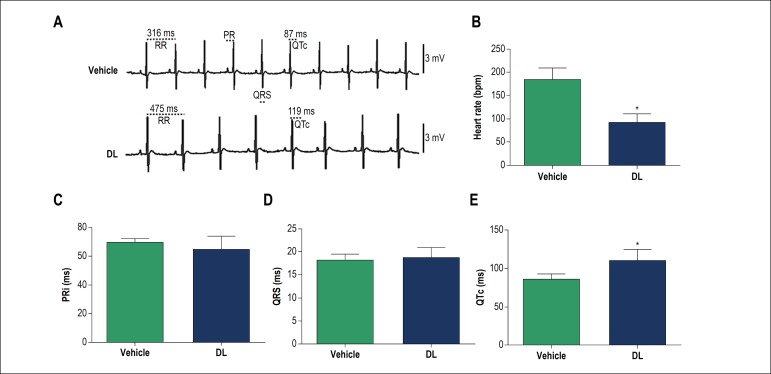
In vivo effects of DL (10 mg/kg) on electrocardiographic parameters (ECG) before (vehicle) and after DL perfusion. Original traces (A) and bar chart of DL effect on heart rate (B), PR interval (PRi) (C), QRS duration (D), and corrected QT interval (QTc) (E). Data are mean ± SD and were analysed by the unpaired Student T test. * p < 0.05 vs vehicle, n = 4.

Figure 3A shows ECG representative tracings for the vehicle (top) and 10 min after i.v. administration of 10 mg/kg of DL (bottom). As can be seen, DL significantly decreased HR (p < 0.05) and increased the QTc (Figures 3B and E). However, DL did not change the PRi and QRS complex duration (Figures 3C and D).

**Figure 4:**
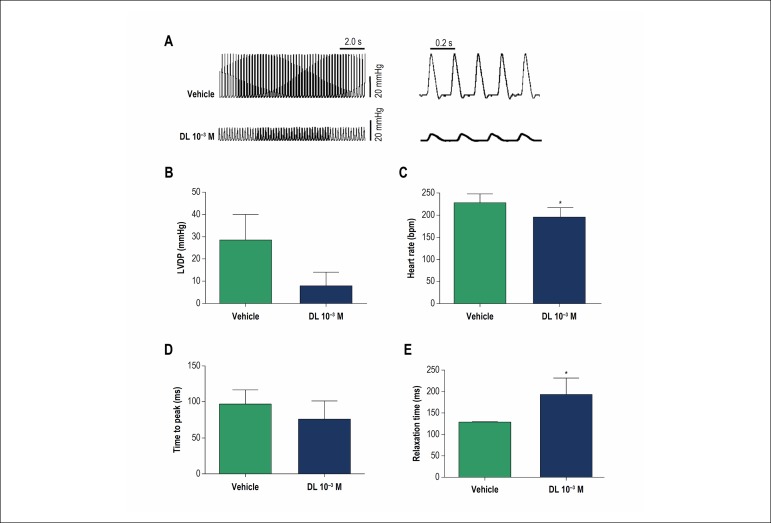
Ex vivo effects of DL perfusion (10^-3^ M) on cardiac parameters. A) Representative traces of left ventricular development pressure (LVDP) recorded in the control situation (top panel) and after perfusion of 10^-3^ M DL (bottom panel); Bar chart graphic of DL effect on LVDP B), heart rate C), Time of peak D), and Relaxation time E). Data are mean ± SD and were analysed by unpaired Student T-test. * p < 0.05 vs vehicle, n = 4.

In the experiment on Langendorff system, DL (10^-3^ M) evoked a significant reduction in LVDP compared to the control (Figure 4A). Average data showed that the LVDP decreased from 25.2 ± 11.4 mmHg to 5.9 ± 6.1 mmHg (n = 5, p < 0.05, Fig 4B). DL also promoted a reduction of HR from 228.6 ± 19.1 to 196.0 ± 20.8 bpm (n = 4, p < 0.05, Figure 4C). Regarding the temporal course of the contraction and relaxation phases, our results showed that DL did not alter the time to peak contraction of the left ventricle (Figure 4D) but increased the relaxation time from 129.0 ± 3.0 to 193.5 ± 37.8 ms (n = 4, p < 0.05, Figure 4E).

**Figure 5:**
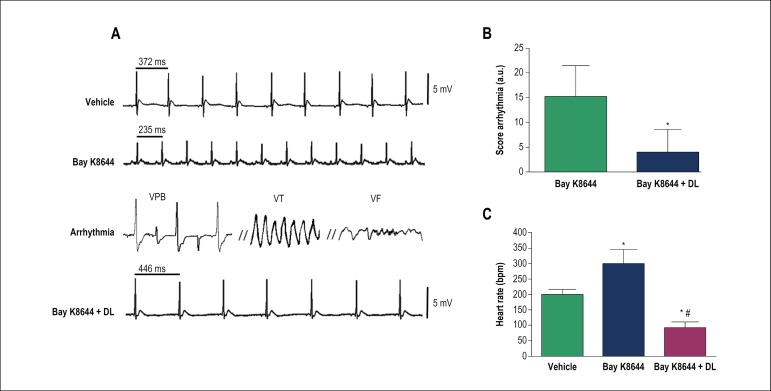
In vivo effects of DL (10 mg/kg) on arrhythmias induced by S(−)-Bay K 8644 in anesthetized rats. Original traces A) and bar chart graphic of arrythmia score B) and heart rate C) before and after DL treatment. Data are mean ± SD and were analysed by unpaired Student T-test (B) (* p < 0,05 vs control, n = 4) and ANOVA followed by Bonferroni post-test (C) (* p < 0,05 vs control, # p < 0.05 vs Bay K8644, n = 4). VPB: ventricular premature beats, VT: ventricular tachycardia; VF: ventricular fibrillation.

In *in vivo* model of arrhythmias induced by Bay K8644, DL significantly reduced the incidence of ventricular arrhythmias, such as VPB, VT and VF, and the total arrhythmia index from 15.3 ± 6.1 a.u to 4.0 ± 4.5 a.u (n = 4, p < 0.05) (Figure 5B). Furthermore, DL was able to prevent the sinus tachycardia evoked by Bay K8644 (300.0 ± 46.1 for 93.0 ± 17.4 bpm, n = 4, p < 0.05).

## Discussion

In conscious rat, the intravenous administration of DL at lower doses (1 and 5 mg/kg) was able to produce a non-significant hypotension, without altering HR. On the other hand, DL at higher doses (10, 20, and 40 mg/kg) produced intense and persistent bradycardia, which persisted up to almost 20 min. Similar effects have been found for other monoterpenes such as 1,8-cineol^17^ terpinen-4-ol,^18^ and y(-)-α-bisabolol.^19^

In order to understand the DL-induced bradycardic effect better, we investigated any changes in the electrocardiographic profile at a dose of 10 mg/kg. This dose was chosen because it was the lowest dose able to produce bradycardia without producing any significant effect on MAP (see Figure 2B). The administration of 10 mg/kg of DL (bottom). significantly decreased HR and increased the QTc. However, DL did not change the PRi and QRS complex duration. Corroborating the data observed in the hemodynamic experiments, we observed in the ECG that DL also promoted bradycardia by increasing QTc. As shown in the literature, calcium channels blockers can reduce cardiac conduction velocity and cause bradycardia.^20,21^ These effects may or may not be associated with QTc prolongation.^22^

The direct effect of DL on the cardiac muscle was evaluated in an isolated heart mounted on a Langendorff system. In this experiment, DL or vehicle was perfused through the aorta to record LVDP. The volume of blood in a rat is approximately 7 mL to each 100 g of body weight,^23^ therefore, the DL concentration after administration of 10 mg/kg in the *in vivo* experiments is approximately the same as the perfusion with 10^-3^ M of DL in the *ex vivo* experiments. As shown, the perfusion with DL evoked a significant reduction in LVDP. Interestingly, these results are very similar to that observed *in vivo*, where DL also promoted a reduction of HR. Regarding the temporal course of the contraction and relaxation phases, our results showed that DL did not alter the time to peak contraction of the left ventricle (Figure 4D) but increased the relaxation time. As known, the recapture of calcium by sarco/endoplasmic reticulum calcium ATPase (SERCA) is a pivotal step for myocardial relaxation in rats ^24^. Our data show that DL delayed the relaxation of cardiac muscle, which can be associated to minor recapture of calcium by SERCA, but this assumption will still need to be elucidated.

Our *ex vivo* results showed that DL decreased pacemaker activity and promoted negative inotropism. It is well known that drugs that block LTCC in cardiac muscle decrease pacemaker activity and result in bradycardia. Furthermore, calcium antagonists decrease myocardial force generation (negative inotropy). These results show that DL presents a similar action to an LTCC blocker. In smooth muscle, De Sousa et al.^25^ showed that the relaxant effects of DL in guinea pig tracheas and rat aortas were independent of the endothelium and probably caused by direct action on calcium dynamics.

LTCC blockers are classified as class IV antiarrhythmic drugs. The antiarrhythmic properties of LTCC blockers are related to their ability to decrease the firing rate of aberrant pacemaker sites within the heart and to their ability to decrease conduction velocity in the AV node that can help to block reentry mechanisms, which can cause supraventricular tachycardia. Indeed, we decided to investigate the possible antiarrhythmic effect of DL using an *in vivo* model with arrhythmia induced by Bay K8644, an LTCC agonist. In all the animals tested, Bay K8644 increased HR and inducing arrhythmias such as VPB, VT and VF. As showed, DL significantly reduced the incidence of ventricular arrhythmias and was able to prevent the sinus tachycardia evoked by Bay K8644.^15^ Similarly to our results, another study showed that verapamil, an LTCC antagonist, was able to prevent the enhancement of total arrhythmia scores induced by Bay K8644. ^15^ These results provide some pharmacological evidence that DL significantly attenuates cardiac arrhythmias evoked by Bay K8644, probably via inhibition of LTCC, however, further experiments should be performed to clarify this assumption.

## Conclusion

The results show that DL has *in vivo* and *in vitro* anti-arrhythmic properties. Furthermore, DL induced bradycardia associated with hypotension in healthy rats (*in vivo*), corroborating the results in an isolated heart (*in vitro*), which DL promoted bradycardia and reduction of left ventricular pressure. We, therefore, consider this monoterpene to be a promising substance to the development of a new therapeutic agents to treat cardiovascular diseases, such as cardiac arrhythmias.
